# Colon Delivery of Budesonide Using Solid Dispersion in Dextran for the Treatment and Secondary Prevention of Ulcerative Colitis in Rat

**Published:** 2010

**Authors:** Jaleh Varshosaz, Fatemeh Ahmadi, Jaber Emami, Naser Tavakoli, Mohsen Minaiyan, Parvin Mahzouni, Farid Dorkoosh

**Affiliations:** 1 Department of Pharmaceutics, Faculty of Pharmacy and Isfahan Pharmaceutical Sciences Research Centre, Isfahan University of Medical Sciences (IUMS), Isfahan, Iran; 2 Department of Pharmacology, Faculty of Pharmacy and Isfahan Pharmaceutical Sciences Research Centre, IUMS, Isfahan, Iran; 3 Department of Pathology, Faculty of Medicine, IUMS, Isfahan, Iran; 4 Department of Pharmaceutics, Faculty of Pharmacy, Tehran University of Medical Sciences, Tehran, Iran

**Keywords:** Budesonide, Solid dispersion, Dextran, Ulcerative colitis, Colon delivery

## Abstract

**Objectives:**

Ulcerative colitis is characterized by local inflammation. Targeting drugs directly to the site of injury has the benefit of lower adverse effects and more effective therapy. The aim of this study was colon targeted delivery of budesonide to deliver the major part of the drug to the colon.

**Methods:**

Matrix tablets of budesonide from solid dispersion of drug with dextran were prepared using different drug to polymer ratios and three molecular weights of dextran. The physical evaluation and drug release behavior were studied. In vivo efficacy of the selected formulation against acetic acid induced colitis in rats was evaluated and compared to the control (untreated) and references (mesalazine and budesonide suspensions) groups.

**Results:**

The results showed that solid dispersion of budesonide with dextran in the ratio of 1:7 using molecular weight (MW) of 10,000 dextran (SDT710) released 25% of the drug in the first 6 hours and 100% in caecal and colonic contents. It could target the drug to colon with improvement in some of the inflammatory signs of induced ulcerative colitis in rat. Treatment with SDT710 could improve not only the percent of involvement also macroscopic damage parameters. The macroscopic parameters included weight/length ratio of the colon, ulcer area, damage score, and ulcer index reduced in comparison to the control group and conventional suspension of budesonide; however, only weight/length ratio was significant.

**Conclusions:**

In the experimental model studied, the new colonic delivery system significantly improved the efficacy of budesonide in the weight/length ratio of the colon in induced colitis in rats.

## INTRODUCTION

Ulcerative colitis and crohn’s disease are two features of inflammatory bowel disease (IBD). They are recognized by chronic relapsing inflammation in the whole GI tract from mouth to anus, but are two distinct entities. Ulcerative colitis is characterized by chronic inflammation in a continuous and confluent pattern which mostly affects rectum and colon.^1^ Corticosteroids are very effective for the treatment of acute flares of IBD and are typically used for moderate-to-severe disease. Treatment with steroids is associated with significant acute and chronic side effects. These include growth retardation in children, osteoporosis, cataracts, hypertension, mood alterations, Cushing syndrome and adrenal atrophy.^1^,^2^ Preparations of corticosteroids in the form of rectal suppositories, enemas and foams have been used. They are used especially when the more distal parts are involved, in order to decrease the side effects of corticosteroids and enhance the efficacy of treatment. Newly developed corticosteroids with high topical activity and low systemic side effects are drugs of choice for treatment of IBD.^1^,^3^,^4^ Budesonide is a potent, synthetic non-halogenated corticosteroid with high topical antiinflammatory effect and little systemic effects. Because of low incidence of corticosteroids adverse effects and high topical effects, budesonide is an important choice for treatment of IBD. Different formulations of budesonide, for example, enemas and controlled release capsules, are now being used for treatment of crohn’s disease.^5^–^7^ The Entocort^®^ capsules release budesonide immediately after oral administration and the drug will be released in the more proximal sites of the small bowel than Budenofalk^®^. Budenofalk is another pH dependent controlled-release formulation of budesonide. With administration of Budenofalk^®^, more budesonide could be delivered in the region of terminal ileum and drug exposure in the systemic circulation is somewhat higher than Entocort^®^. These preparations seem unable to deliver sufficient drug to the distal colon and rectum where the drug is needed in ulcerative colitis. The treatment of colon diseases such as ulcerative colitis, colorectal cancer and crohn’s disease is more effective with direct delivery of drugs to the affected area. In order to improve the efficacy of budesonide for treatment of ulcerative colitis, different delivery systems controlling release of budesonide in the distal ileum to the sigmoid colon have been developed.^8^–^10^ Solid dispersion of drugs in a biodegradable polymer could be a new method for specific drug delivery to the colon. By using a suitable polymer, for example, a polysaccharide which is not soluble in the stomach and small intestine, the drug solubility could be manipulated. Drug solubility in the upper part of the GI tract could be controlled by the solubility properties of the polymer and after reaching to the colon, the polymer is degraded by the colonic microflora and the drug is released into the colon.

Solid dispersion technology has been applied for the controlled release of drugs. The structure of the solid dispersion is monolithic where drug molecules homogeneously disperse. It has a great advantage for avoiding the risk of a burst release feared concerning the reservoir type preparations. By using polysaccharide carriers which are degraded specifically by the colonic microflora, the drug release could be controlled in the stomach and small intestine. Dextran is a polysaccharide which is suitable for colon drug delivery, especially the high molecular weight types which are less soluble in the aqueous media. Previous studies^11^ on the chemical conjugation of budesonide with dextran using hemisuccinate spacer showed promising results as a prodrug for colon specific delivery of budesonide.

### Physical evaluation of tablets prepared from solid dispersions

Hardness, weight variation and content uniformity of tablets were determined according to pharmacopeial standard tests.^13^

Further pathophysiological studies on animals performed in our lab proved its effectiveness as a new formulation for treatment of ulcerative colitis. The aim of this study was colon targeted delivery of budesonide to deliver the major part of the drug to the colon using physical approach of solid dispersion technique instead of chemical prodrug preparation. This approach can reduce side effects of the drug and increase its effectiveness in the site of action. Both of these can cause reduction of the needed dose and consequently, better tolerance of drug in secondary prevention of progression of the disease.

## METHODS

### Materials

Budesonide was provided by AstraZeneca (UK). Magnesium stearate, Acetone, KH_2_ PO_4_ and NaH_2_ PO_4_ were purchased from Merck (Germany). HPLC grade acetonitrile and methanol were supplied by Caledon (Canada). Dextrans (of different molecular weights 10000, 70000 and 500000) were purchased from Sigma (St. Louis, USA) and Avicel provided by Fluka (Germany). All other solvents and chemicals were of analytical grade.

### Preparation of budesonide-dextran solid dispersions

Budesonide-dextran solid dispersions were prepared by solvent deposition technique.^12^ Budesonide was dissolved in acetone to produce a clear solution. Then, dextran of three molecular weights was dispersed in the solution by stirring at room temperature for 15 h. The solvent was removed in rotary evaporator at 60°C. The resulting mass was dried at 40°C for 24 h, pulverized and passed through a sieve with a mesh number 40. Three different ratios of drug to polymer, namely 1:4, 1:7 and 1:10 were used for solid dispersion formulations.

### Preparation of tablets of solid dispersions

The resulting solid dispersions were mixed with Avicel (20 min) and then, magnesium stearate (5 min) and pressed to tablets using a single punch tabletting machine (KS43373-202, Killian Co., Germany) with flat faced punches with 5 mm diameter. Each tablet (average weight 50 mg for 1:4 and 1:7 ratios of drug to polymer and 70 mg for 1:10 ratio of drug to polymer) contained 3 mg budesonide. The details of formulations are presented in Table 1.

**Table 1:** Formulation details of prepared tablets using solid dispersion of budesonide and dextran

Formulation code *	Dextran MW	Dextran amount (mg)	Budesonide (mg)	Avicel (mg)	Avicel (mg)
SDT410	10000	12	3	35.5	0.5
SDT470	70000	12	3	35.5	0.5
SDT4500	500000	12	3	35.5	0.5
SDT710	10000	21	3	25.5	0.5
SDT770	70000	21	3	25.5	0.5
SDT7500	500000	21	3	25.5	0.5
SDT1010	10000	30	3	36.3	0.7
SDT1070	70000	30	3	36.3	0.7
SDT10500	500000	30	3	36.3	0.7

* The first digit in the code stands for the ratio of drug to polymer and the second one presents the molecular weight (MW) of dextran (KDa)

### Release studies of budesonide after incubation with rat GI contents

Within one week prior to the start of the dissolution studies, male Wistar rats were maintained on normal diet and 1 ml per day of 2% w/v solution of dextran in water was administered directly into the stomach in order to induce enzymes specifically acting on dextran in the caecum and colon. The rats were then sacrificed by decapitation and after midline incision, luminal contents of stomach, small intestine, caecum and colon were removed and transferred to appropriate buffer solutions containing 0.5% sodium lauryl sulfate (SLS) to preserve the sink condition for budesonide. Contents were homogenated in HCl 0.1 N containing 0.5% SLS for 2 h (to simulate the gastric pH 1.2), phosphate buffer solution (PBS) (pH 7.4) containing 0.5% SLS for 4 h (to simulate small intestinal pH) and PBS (pH 6.8) containing 0.5% SLS and 4% rat caecal and colonic contents for 18 h (to simulate colonic environment of rat). These are the reported normal pH values of rat GI tract.^8^ The phosphate buffer solution used to dilute caecal and colonic contents was saturated with N_2_ to maintain the anaerobic condition. Drug release studies were performed in triplicate on each conjugate (an amount equivalent to 3 mg budesonide) in 50 ml appropriate buffer solution at 37°C using an undersized, homely designed USP dissolution apparatus II (paddle method). At predetermined time intervals, 100 μl sample of each medium was withdrawn and replaced with the same volume of fresh medium. After addition of methanolic solution of the internal standard, the samples were vortexed and centrifuged at 10000 rpm for 5 min and 50 μl of the supernatant was injected into the HPLC.

### Determination of drug in formulations and dissolution samples

A reversed-phase HPLC method was used for determination of budesonide. HPLC analysis was performed using a Waters 515 pump; Waters 2487 dual λ absorbance detector and data were integrated using Millennium^®^ software for HPLC. A C_18_ Waters μ-Bondapak HPLC column (250 × 4.6 mm) and a mobile phase consisting of acetonitrile: KH_2_ PO_4_ 0.025 M (55:45, pH 3.2) at a flow rate of 1 ml/min were applied. The eluent was detected at 244 nm. Injection volume was 50 μl and dexamethasone was applied as an internal standard. Quantitation was achieved by measurement the peak area ratios of the drug to the internal standard. The mobile phase was prepared daily, filtered and degassed by ultrasonication before use.

### In vivo studies

#### Animals

Male Wistar rats (200 ± 20 g) from animal house of the faculty of Pharmacy of Isfahan University of Medical Sciences were used. They were housed in environmentally controlled conditions (22 ± 2°C, 12-h light–dark cycle), with free access to water and standard chow pellet diet. The rats were allowed to acclimatize for 1 week before experiment. They were fasted for 36 h before induction of colitis. The animal studies were carried out according to the guidelines of the ethical committee of Isfahan University of Medical Sciences.

#### Induction of colitis

Induction of colitis was performed according to the method described in previous studies.^14^,^15^ Under light ether anaesthesia, a polyethylene tube with 8 cm length, was inserted into the anus and 2 ml of a 4% acetic acid solution was instilled to the colon. The animals were left with free access to pellets and water until recovery.

#### Administration of drugs

Tablets for administration to animals were prepared using a 2 mm flat faced punch in a single punch tabletting machine and administered via a NG tube (No. 8) fixed on a feeding tube No. 18. The rats were divided into 5 groups of 6 as follows:

1. Normal or sham group (without induction of colitis) which received 2 ml normal saline via rectal route.
2. Control group which received placebo dextran tablets (without drug) orally.
3. Reference group I which received mesalazine suspension in 1% (w/v) carboxymethyl cellulose (CMC) solution (120 mg/kg) orally.
4. Reference group II which received budesonide suspension in 1% (w/v) CMC solution (300 μg/kg) orally.
5. Test group received selected tablet formulation of budesonide-dextran solid dispersions, SDT710 (300 μg/kg).

Drug administration to animals was started 24 h after induction of colitis and repeated every 24 h for 5 days.

### Assessment of colonic injury and inflammation

Twenty four h after administration of the last dose of formulations, rats were sacrificed with high dose of ether and a midline incision was made in abdomen. The 8-cm distal segment of colon was removed, opened and washed in normal saline, weighed, and the colon wet weight/length (mg/cm) ratios obtained as a criterion of injury. Severity of gross macroscopic injury was assessed using a scoring system reported previously^16^ with a slight modification as follows: 0, normal appearance; 1, erythema and inflammation without ulcer; 2, inflammation and ulcer; 3, ulcer with necrosis. Inflammation and ulcer surface area was measured and the ulcer index was calculated by following equation:^17^

[eq. 1] $$\mathrm{Ulcer}\;\mathrm{Index}\;=\;\mathrm{Ulcer}\;\mathrm{area}\;\left(cm^{2}\right)\;+\;\mathrm{Macroscopic}\;\mathrm{score}\;$$

Sections of colon specimens were fixed in phosphate-buffered formalin solution (10%), embedded in paraffin, stained with haematoxylin and eosin (H and E) and evaluated by light microscopy for morphological changes. Inflammation extent and severity, crypt damage and percent of involvement were considered to assess the colonic damage from the histopathological point of view.^18^ A scale was defined for each of the four criteria based on the percent of damage which is presented in Table 2.^19^ Total colitis was also calculated by summation the scores of inflammation severity, inflammation extent and crypt damage.

**Table 2:** Scoring system for pathological assessment of colitis^19^

Scoring parameter	Score definition
Inflammation severity	0: None
	1: Mild
	2: Moderate
	3: Severe
Inflammation extent	0: None
	1: Mucosa
	2: Mucosa and submucosa
	3: Transmural
Crypt damage	0: None
	1: Basal 1/3 damaged
	2: Basal 2/3 damaged
	3: Crypts lost, surface epithelium present
	4: Crypts lost, surface epithelium lost
Percent of involvement	0: 0%
	1: 1-25%
	2: 26-50%
	3: 51-75%
	4: 76-100%

### Statistical analysis

Kolmogorov-Smirnov statistical test was used for controlling normality and homogeneity of the data. Differences between mean values were analyzed using one-way analysis of variance (ANOVA) followed by a Dunnett’s post-hoc test or Kruskal-Wallis test when appropriate (SPSS 11.5). P-values less than 0.05 were considered significant for all statistical tests.

## RESULTS

### Physical evaluation of tablets

All examined formulations gave tablets with good and reproducible technological properties; i.e., acceptable weight variation [coefficient variation (CV%) <2%] and hardness which was in the recommended range by official references.^13^,^20^ Drug content distribution of each for-mulation was uniform and CV% of the drug amount of all formulations was less than 6%. According to USP (13), drug content of the tablet should be in the range of 85-115% and the CV% less than 6%.

### Release studies of tablets prepared from dextran-budesonide solid dispersion

**Figure 1:**
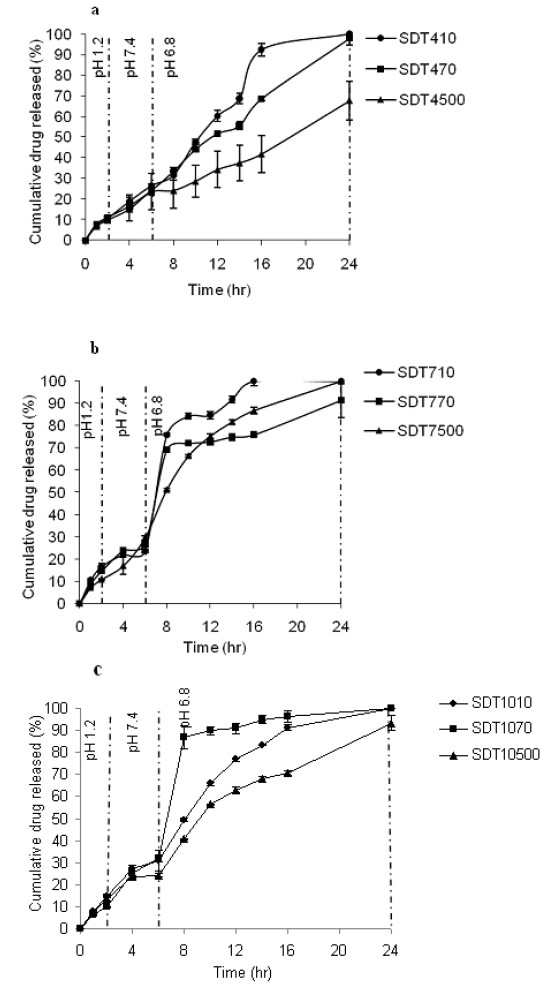
Release profiles of budesonide from tablets of solid dispersions with a)1:4, b) 1:7 and c) 1:10 drug to dextran ratio in the presence of rat caecal and colonic contents

Figure 1a shows the release profiles of tablets prepared from solid dispersed particles with 1:4 drug to polymer ratio. The drug release profiles of SDT4 tablets in Figure 1a show that only in the case of SDT4500, the drug release rate was slow and the overall percentage of drug released was only 68%. For SD tablets containing 1:7 drug to polymer ratio (SDT7 tablets) the pattern of release was similar to SDT4 tablets except that in this case, the release was sharply increased after addition of rat colonic contents that shows the susceptibility of this formulation to the microflora of colon (Figure 1b). For SD tablets containing 1:10 drug to polymer ratio (SDT10 tablets), release profiles in the presence of rat caecal and colonic contents are shown in Figure 1c. As can be concluded from Figure 1, SDT710 releases almost the entire amount of drug loaded in the colon environment while the least amount of drug is released in acidic and intestinal medium (less than 20%). Although this pattern may be seen in some other formulations like SDT410 but as mentioned earlier, SDT710 was more susceptible to bacterial degradation. This was concluded from the sharp change of drug release pattern by changing the pH and microflora of colonic medium.

### In vivo studies

#### Macroscopic assessment of the colon

Colon wet weight/length ratio, ulcer area, ulcer index and macroscopic damage score were

the parameters investigated for macroscopic assessment of the colon.Table 3 summarizes the data of macroscopic evaluation of colon damage of normal, control and treatment groups. Animals experienced diarrhea and weight loss after instillation of acetic acid solution. Macroscopic sectioning showed signs of inflammation, haemorrhage and ulcers and bowel wall thickening which was evident in control group of rats with a significant increase in the colonic weight/length ratio in comparison to non-colitic rats (Table 3). Ulcer area, ulcer index and macroscopic damage score were also statistically different between normal and control groups. All the treatment groups except mesalazine group attenuated the ulcer area, ulcer index and macroscopic damage score, however, the difference was not significant compared to the control group.

**Table 3:** Data of macroscopic evaluation of colitis of different treatment groups after 5 day treatment

Treatment group	Macroscopic damage parameters			
	Weight/length ratio (mg/cm)	Ulcer area (cm^2^)	Damage score**	Ulcer index
Normal	67.1 ± 5.7	0.0 ± 0.0	0	0.0 ± 0.0
Control	165.9 ± 12.7	1.6 ± 0.6	2	3.8 ± 0.9
Mesalazine	160.3 ± 14.3	1.7 ± 0.5	2	3.5 ± 0.9
Budesonide	137.2 ± 11.7	1.0 ± 0.3	2	2.6 ± 0.8
SDT710	117.7 ± 5.2*	1.2 ± 0.2	1	2.6 ± 0.5

All values are reported as mean ± SEM (n=6)

* P<0.05 compared to the control.

** Values are presented as median of the data.

#### Histological assessment of the colon

**Figure 2:**
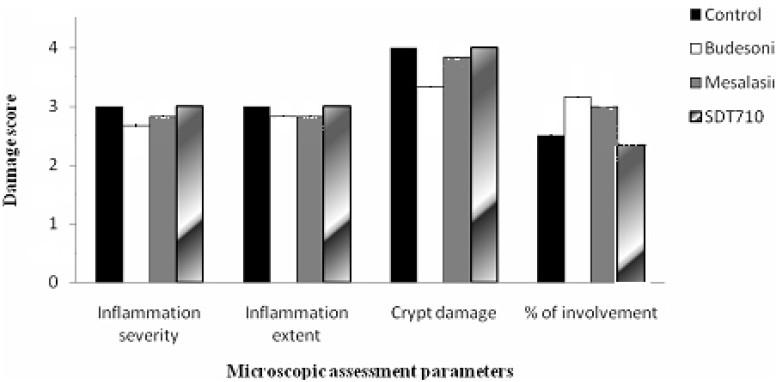
Comparison of the pathological scores of colitis in control and treatment groups after 5day treatment (n=6)

Figure 2 illustrates the effects of treatment on the pathological scores of induced colitis. No significant difference was seen between studied groups in crypt damage and the inflammation severity or extent. However, the percent of involvement was significantly different (P<0.05) in the groups treated with SDT710 and control group with the other two groups.

## DISCUSSION

Since dextran cannot be melted easily and the drug and polymer could not be dissolved in a common solvent, solvent deposition method was applied for preparing dextran-budesonide solid dispersions. The drug was dissolved in a suitable solvent and then, the polymer was dispersed in it. Then, the solvent was evaporated and the residue was the solid dispersion of drug in the polymer. Solid dispersions prepared by this method were pressed to tablets. Considering the final dosage form of the solid dispersion particles, it is desirable to select the tablet among the various types of dosage form, because of its convenience in production and usage. Tablet has various advantages, such as portability, patient compliance and lower cost in production compared with other solid dosage forms.^12^ Dissolution studies were performed in the three mentioned media to show the colon target ability of the developed formulations. MW of the polymer and drug to polymer ratio were two effective parameters on the drug release. The dependency of drug dissolution to the ratio of drug to polymer is due to the greater reduction in drug diffusion into the medium which was also observed for dimenhydrinateethyl cellulose solid dispersions.^21^ Dissolution data showed that SD tablets prepared by MW 500000 could protect the drug from premature release in stomach and small intestine since the aqueous solubility of this MW of the polymer is very low and its rate of dissolution is slow. Therefore, the SD prepared by this MW, could delay the drug release until the formulation reaches to the colonic contents. Inversely for lower MWs, (i.e., 10000 and 70000), SD preparation has increased the aqueous solubility of budesonide as dextran 10000 and 70000 are very soluble in water and cannot protect the formulation in HCl 0.1 N and phosphate buffer pH 7.4. The higher dissolution rate of SD formulations of dextran 10000 and 70000 relative to pure drug is probably due to adsorption of the hydrophilic particles of dextran onto the hydrophobic budesonide particles, which in turn might enhance the wet ability of the latter particles. The drug dissolution is increased considerably from the SD formulations and this increase, strongly depends on the ratio of drug to carrier. The similar results have been obtained for SD formulations of glibenclamide with Avicel.^22^ Dissolution study of SD tablets containing MW 500000 of the polymer were also performed in the absence of colonic contents and the results showed that there was no significant difference in the release profiles of these formulations in the presence and absence of colonic contents (data not shown). This study proved that the release of drug from SD formulations was related to solubility of the polymer and movement of the dissolution media and enzymatic degradation of the polymer didn’t play a significant role. Although the formulation SDT4500 was successful at controlling the release in HCl 0.1N and phosphate buffer pH 7.4, it didn’t release the drug completely after 24 h.

Other formulations also were not so efficient except SDT710. It may be because of the high water solubility of low molecular weight dextrans. For better results, probably very high molecular weights of dextran, for example higher than 5000000 should be applied. In a study, a very high molecular weight dextran (about 5000000) was used for preparing sustained release formulation of propranolol and it was effective to release the drug in 24 h.^23^ Among the studied formulations, only SDT710 released 24% of the drug in the first 6 h and all the drug content after 24 h. The release profile of this formulation showed a sharp increase (about 50%) after exposing to the caecal and colonic contents (Figure 1). So, this formulation was selected as the optimum formulation between studied formulations for *in vivo* studies. The wet weight of the inflamed colon tissue is considered as a reliable and sensitive indicator of the severity and extent of inflammatory response.^24^ The tested formulations reduced the wet weight of distal colon segments and the colon damage score, compared with controls that received the vehicle. In the case of colon wet weight/length ratio, the difference was significant only for the group treated with SDT710. The efficacy of SDT710 formulation in reducing macroscopic damage score was higher than budesonide and mesalasine suspensions (Table 3). This observation showed that the concentration of budesonide delivered specifically to the colon by this formulation was higher. The observation in histological assessment of the colon showed that treatment with mesalasine could not attenuate the histological intensity of colitis. The similar pattern was observed for the group of rats treated with budesonide suspension. Treatment with budesonide suspension could not develop any significant change in the histological score of colitis in comparison to the control group. Pathologic scores of the group treated with SDT710 showed significant reduction in the percent of involvement compared to other groups.

## CONCLUSIONS

The findings confirmed that the administration of tablet formulation of solid dispersion of budesonide with dextran in the ratio of 1:7 and using molecular weight of 10000 of dextran (SDT710) may represent an effective tool for the treatment of colonic inflammatory bowel disease. This colonic delivery system caused a significant decrease in inflammation in the colon of colitic rats after oral administration, compared with the same dose of the drug administered as an oral suspension. The results allow for the conclusion that in the experimental model studied, the new colonic delivery system significantly improved the efficacy of budesonide in the macroscopic healing of induced colitis in rats (the colonic weight/length ratio). To enhance the pathologic scores of colitis, better coverage of budesonide particles by dextran seems necessary to increase availability of the drug to the affected area of the colon. Coating the drug particles with this polymer by spray drying technique is suggested. The described system may be more useful than budesonide itself for clinical treatment and prevention of the development of colonic inflammatory bowel disease.
